# Hypofibrinolysis in pediatric patients with veno-occlusive disease in hematopoietic stem cell transplantation

**DOI:** 10.1007/s00432-023-04798-9

**Published:** 2023-04-22

**Authors:** Veronika Schneider, Karin M. Cabanillas Stanchi, Karina Althaus, Sarah Schober, Sebastian Michaelis, Christian Seitz, Peter Lang, Rupert Handgretinger, Tamam Bakchoul, Stefanie Hammer, Michaela Döring

**Affiliations:** 1 Department I–General Pediatrics, Hematology and Oncology, University Children’s Hospital Tübingen, Hoppe-Seyler-Str. 1, 72076 Tübingen, Germany; 2 Center for Clinical Transfusion Medicine, University Hospital of Tübingen, Tübingen, Germany; 3 Institute for Clinical and Experimental Transfusion Medicine, Medical Faculty of Tübingen, Tübingen, Germany

**Keywords:** Thrombelastography, Veno-occlusive disease, Hematopoietic stem cell transplantation, Hypofibrinolysis, Viscoelastic tests

## Abstract

**Purpose:**

Veno-occlusive disease (VOD) is a serious complication of hematopoietic stem cell transplantation (HSCT) with a high incidence in pediatric patients. This study aimed to detect signs of hypofibrinolysis using thrombelastography.

**Methods:**

In this prospective single-center study, thrombelastographic measurements (EX and TPA tests) were taken during HSCT to detect signs of impaired coagulation, clot formation, or hypofibrinolysis.

**Results:**

Of 51 patients undergoing allogeneic and autologous HSCT, five (9.8%) developed VOD and received defibrotide treatment. Thrombelastography measurements were also obtained from 55 healthy children as a control group. The results show that clot lysis was prolonged in VOD patients compared to other HSCT patients and control group (lysis time, TPA test: day + 14 to + 21: VOD: 330 ± 67 s vs. HSCT: 246 ± 53 s; *p* = 0.0106; control: 234 ± 50 s; control vs. VOD: *p* = 0.0299). The maximum lysis was reduced in HSCT patients compared to controls (EX test: control: 8.3 ± 3.2%; HSCT: day 0 to + 6: 5.3 ± 2.6%, *p* < 0.0001; day + 7 to + 13: 3.9 ± 2.1%, *p* < 0.0001; day + 14 to d + 21: 4.1 ± 2.3%, *p* < 0.0001).

**Conclusion:**

These results suggest that HSCT patients exhibit reduced fibrinolytic capacities and patients diagnosed with VOD show signs of hypofibrinolysis. This prospective study shows that fibrinolysis can be assessed in a rapid and accessible way via thrombelastography. Thrombelastography might be a parameter to support the diagnosis of a VOD and to serve as a follow-up parameter after the diagnosis of a VOD.

## Introduction

Veno-occlusive disease (VOD) or sinusoidal obstruction syndrome (SOS) is a rare but serious complication in patients undergoing hematopoietic stem cell transplantation (HSCT), whereby especially pediatric patients are at an increased risk. The reported incidence for children and adolescents undergoing allogeneic or autologous HSCT is 11–27%, while for adult patients it is 7–15% (Barker et al. 2003; Carreras et al. 2011; Cesaro et al. 2005; Coppell et al. 2010; Kalayoglu-Besisik et al. 2005; Lee et al. 2010; Reiss et al. 2002). VOD plays an important role in HSCT-associated morbidity and mortality. Mortality on day + 100 after HSCT is 6–9.5% in children without VOD and 23–38.5% in those who develop VOD (Barker et al. 2003; Reiss et al. 2002). Even though the detailed pathophysiology remains to be elucidated, current pathophysiological concepts point to VOD as a result of hepatic endothelial damage with increased (local) coagulability and hypofibrinolysis leading to obstruction and fibrosis of hepatic sinusoids. This induces portal hypertension, which causes consequent clinical symptoms (Cairo et al. 2020). In clinical practice, VOD in children is diagnosed with criteria defined by the European Society for Blood and Marrow Transplantation (EBMT) (Corbacioglu et al. 2018). These criteria include weight gain, ascites, hepatomegaly, hyperbilirubinemia, and refractory thrombocytopenia. To date, there are no established markers or tools for an earlier diagnosis of VOD in common clinical practice. In addition to the best supportive care, which includes optimized fluid management and diuretics, VOD is treated with the oligonucleotide defibrotide which has been shown to reduce endothelial inflammation and adhesion as well as to reduce thrombosis and enhance fibrinolysis (Pescador et al. 2013). The early initiation of defibrotide treatment improves the probability of complete remission of VOD and survival (Corbacioglu et al. 2004; Kernan et al. 2018).

Thrombelastography is a readily available tool to assess coagulation, clotting and clot lysis as a point of care testing. It is used in the field of trauma management, cardiac surgery, and to manage bleeding disorders (Whiting and DiNardo 2014). In the context of HSCT, impaired clot formation was observed via thrombelastography in adult patients undergoing HSCT and developing VOD (Rupa-Matysek et al. 2017). Another group evaluated abnormal routine coagulation tests in pediatric VOD patients and controlled substitution of coagulation factors via thrombelastography (Gendreau et al. 2017).

Until now, the aspect of assessing hypofibrinolysis, which plays a role in VOD pathogenesis, via thrombelastography has not been elucidated. This study aimed to detect signs of hypofibrinolysis in HSCT and VOD patients, to evaluate changes in thrombelastography parameters during defibrotide treatment, and to discover correlations between altered thrombelastography-values and established symptoms and risk factors of VOD.

## Materials and methods

### Ethics

This prospective single-center study was performed in accordance with the Helsinki Declaration of 1964, as revised in 2013, and with the standards of the institutional ethics committee (No. 109/2021BO1).

### Study design

Patients admitted for HSCT to the pediatric bone marrow transplantation ward at the University Children’s Hospital Tübingen, Germany, between May 2020 and November 2021 were included in the study. The exclusion criteria were admission to therapies other than HSCT and multiple transplants during the study period. The observation period was the time between admission and discharge from the ward. Patients who developed VOD will further be referred to as the VOD group, while those who did not develop VOD as the HSCT group.

The control group patients underwent the placement of an intravenous cannula prior to minor surgical procedures or follow-up blood tests years after recovering from oncological diseases and were otherwise healthy.

### Blood sampling and laboratory analyses

Blood samples (3 ml of citrated whole blood) for thrombelastography were taken once to twice weekly in addition to daily routine blood tests between 6 and 7 a.m. Routine testing included flow cytometric blood counts, coagulation parameters, D-dimers, and bilirubin. To evaluate the symptoms of VOD, patients were examined daily by a physician and their body weight was measured twice a day. Ultrasound screenings for ascites, progredient hepatomegaly, and portal venous flow velocity were performed in case of suspected VOD.

A total of 6 ml of citrated whole blood were collected from the control group, of which 3 ml were used for routine coagulation testing and 3 ml for thrombelastography.

### Thrombelastography

Thrombelastography was performed at the Center for Clinical Transfusion Medicine, University Hospital of Tübingen. Same-day measurements were obtained with the ClotPro device (ClotPro Enicor GmbH, Munich, Germany) for elastic motion thrombelastography. In this system, the cup holding the sample of citrated whole blood rotates around a fixed pin. The process of coagulation and clot formation is recorded over time by assessing the increasing resistance of the clot against the rotational movement. EX and TPA tests were performed. In both tests, the coagulation is initiated by a recombinant tissue factor. Additionally, during the TPA test, the fibrinolysis is activated by a recombinant tissue plasminogen activator. The coagulation time (CT), clot formation time (CFT), clot amplitude after 5 min (A05), maximum clot firmness (MCF), maximum lysis (ML), α-angle, and lysis time (LT) were measured.

### Statistics

The day of HSCT is referred to as day 0, the days before HSCT are counted negatively and days after HSCT positively. Samples were grouped into four categories: day (d)-7 to d-1, d0 to d + 6, d + 7 to d + 13, and d + 14 to d + 21. As the data were not normally distributed, non-parametric tests were used. Two groups were compared with the Mann–Whitney-U-Test and multiple groups with the Kruskal–Wallis- and Dunn’s multiple comparison test. Pearson’s and Spearman’s Rank Correlation were calculated for correlation analyses. Tests were performed with GraphPad Prism for Windows, version 9.3. (GraphPad Software, San Diego, CA, USA). *P*-values of *p* < 0.05(*), *p* < 0.01(**), *p* < 0.001(***), and *p* < 0.0001(****) were defined as statistically significant.

## Results

### Patient characteristics

A total of 51 patients with a median age of 7.8 years (range: 9 months–19 years) were included. The patient characteristics of both study cohorts are summarized in Table 1. The control group consisted of 55 children and the differences in age between the study and the control cohorts were not significant (*p* = 0.53), although the study cohort enrolled significantly more female patients (31 vs. 19 patients, *p* = 0.007). The underlying diseases were mostly malignant (40 patients, 78%), 13 patients suffered from acute lymphoblastic leukemia (ALL) (25%), eight from neuroblastoma (16%), and six patients from acute myeloid leukemia (AML) (12%). Depending on the type of HSCT, different conditioning regimens were used. Of the patients receiving autologous HSCT (*n* = 15; 29%), one (2%) received only busulfan, and four (8%) busulfan and melphalan. Chemotherapeutic conditioning for allogeneic HSCT (*n* = 36; 71%) was either busulfan-based with fludarabine and thiotepa or cyclophosphamide and thiotepa (*n* = 5; 10%), or based on treosulfan or melphalan with fludarabine and thiotepa (*n* = 18; 36%). Total body irradiation (TBI) and etoposide were used in 11 patients (22%) before allogeneic HSCT. All patients received intravenous anticoagulation with 100 international units per kilogram per day of unfractionated heparin as a 24-h infusion. Prophylactic defibrotide was administered to one (2%) patient. The median observation period was 45 days (range 25–176 days). No deaths occurred during the observation period.

**Table 1:** Patient characteristics

Characteristic	*n,* study group	(%)	*n,* control group	(%)	*p*
Total	51	(100)	55	(100)	
Gender					
Female/Male	31/20	(60/40)	19/36	(35/65)	*p* = 0.007
Age group					
≤ 6 yr	23	(45)	30	(55)	
7–11 yr	16	(31)	14	(25)	
12–18 yr	10	(20)	8	(15)	
≥ 18 yr	2	(4)	3	(5)	
Median age	7.8 years		6.7 years		*p* = 0.53
Range	9 months–19 years		3–32 years		

Characteristic	*n,* study group	(%)	Characteristic	*n,* study group	(%)
Total	51	(100)	VOD	5	(10)
Donor			Moderate	2	(4)
Autologous	15	(29)	Severe/very severe	3	(6)
MUD	20	(39)	Defibrotide	6	(12)
MFD	7	(14)	Conditioning		
MMFD	9	(18)	Bu (autologous)	1	(2)
Primary diagnosis			Bu + Mel (autologous)	4	(8)
Acute lymphoblastic leukemia	13	(25)	Bu + Flud + TT or Bu + Mel + Cy (MFD/MUD)	5	(10)
Acute myeloid leukemia	6	(12)	Treo/Mel + Flud + (TT) (MFD/MUD)	9	(18)
Chronic myeloid leukemia	1	(2)	Treo/Mel + Flud + (TT) (MMFD)	9	(18)
Myelodysplastic syndromes	1	(2)	TBI + Etoposide	11	(22)
β-Thalassemia major	2	(4)	Other conditioning	12	(24)
Sickle cell anaemia	3	(6)	GvHD prophylaxis		
Lymphoma	3	(6)	Thymoglobulin^®^	5	(10)
Neuroblastoma	8	(16)	Grafalon^®^	27	(53)
Other solid tumors	8	(16)	CsA	1	(2)
Severe aplastic anaemia	1	(2)	CsA + MTX	23	(45)
Autoimmune disease	1	(2)	Other (MMF, Tacrolimus)	11	(22)
Congenital thrombocytopenia	1	(2)	None	16	(31)
Congenital dyserythropoietic anemia	1	(2)	Previous treatment		
Kostmann’s disease	1	(2)	Gemtuzumab-Ozogamicin	2	(4)
Metachromatic leukodystrophy	1	(2)	Death during observation period	0	(0)

*Bu* busulfan, *CSA* ciclosporin A, *Cy* cyclophosphamide, *Flud* fludarabine, *GvHD* graft-versus-host disease, *Mel* melphalan, *MFD* matched family donor (sibling), *MMF* mycophenolate mofetil, *MMFD* mismatched family donor (haploidentical), *MTX* methotrexate, *MUD* matched unrelated donor, *n* sample size, *p* probability value, *SCID* severe combined immunodeficiency, *TBI* total body irradiation, *TT* thiotepa, *Treo* treosulfan, *VOD* veno-occlusive disease, *yr* years of age

### VOD patients

Of 51 patients, five (10%) were diagnosed with VOD according to the EBMT criteria (Corbacioglu et al. 2018). The VOD was graded moderate (*n* = 2; 4%), severe (*n* = 1; 2%) or very severe (*n* = 2; 4%). Defibrotide treatment was initiated for 4 weeks or until the resolution of symptoms in standard dosing (25 mg per kilogram per day (mg/kg/d)) in four doses over 2 h). The median duration of treatment was 37 days (range 24 to 52) and treatment commenced at a median of + 2 days (range −20 to 51) after HSCT.

VOD patient 1, a 19-year-old young woman, underwent allogeneic HSCT for relapsed ALL. The conditioning consisted of melphalan, fludarabine, and anti-thymocyte globulin (ATG) (Grafalon^®^). Due to T-cell receptor αβ depletion of the graft, no further graft-versus-host-disease (GvHD) prophylaxis was given. VOD was diagnosed on d + 4 with weight gain, ascites, progredient hepatomegaly, and thrombocytopenia but only mild hyperbilirubinemia. Ultrasound showed reduced portal venous flow velocity (minimum 12.5 cm per second (cm/s)). The VOD was graded as very severe due to refractory ascites with necessity for paracentesis and refractory thrombocytopenia lasting several weeks. Defibrotide was administered for 52 days. After delayed response with persistent ascites, defibrotide dosing was increased to 40 mg/kg/d. The portal venous flow velocity normalized in ultrasound controls after 5 days of treatment, ascites diminished after 40 days, and hepatomegaly was also regressive.

VOD patient 2, a nine-year-old girl, received haploidentical HSCT due to myelodysplasia-related AML as a secondary malignancy after nephroblastoma. Conditioning with melphalan, fludarabine, thiotepa, and ATG (Grafalon^®^) started directly following a course of 5-azacytidine and venetoclax. The GvHD prophylaxis consisted of mycophenolate mofetil (MMF). Diagnosis of VOD was established early during the conditioning (d-10) with rising hyperbilirubinemia (total bilirubin: maximum 4.6 mg/dl), weight gain, thrombocytopenia and progredient hepatomegaly. The grading was very severe due to high bilirubin levels and persistent refractory thrombocytopenia. The patient was treated with defibrotide for 37 days. Hyperbilirubinemia was regredient after 14 days of treatment, while thrombocytopenia with daily substitutions lasted for 20 days.

VOD patient 3, a 10-year-old girl, received haploidentical HSCT due to relapsed AML. The patient had already suffered from VOD in a first HSCT 3 years earlier. Before HSCT, the patient received therapy with gemtuzumab ozogamicin, during which she developed VOD with weight gain, ascites, and hepatomegaly. Furthermore, altered portal venous perfusion was observed. Defibrotide was started 20 days prior to HSCT and continued for 52 days. The symptoms of VOD worsened during HSCT and included increasing ascites, further reduction of portal venous flow velocity (minimum 11 cm/s, d + 9) and progredient painful hepatomegaly. The grading of VOD was severe due to altered coagulation. Conditioning was performed with melphalan, fludarabine, thiotepa, and ATG (Grafalon^®^). For GvHD prophylaxis MMF was administered. The portal venous flow normalized 14 days after HSCT (33 days of defibrotide). Hepatomegaly was regressive and ascites disappeared after 39 days of treatment.

VOD patient 4, a 14-year-old boy, suffered from ALL and received allogeneic HSCT. Conditioning started shortly after chemotherapy with a triple course consisting of clofarabine, cyclophosphamide, and etoposide due to non-remission. After cell regeneration, a conditioning with TBI (12 Gray), etoposide, and ATG (Grafalon^®^) was performed. For GvHD prophylaxis, he received cyclosporin A (CsA) and methotrexate (MTX). Due to the development of renal insufficiency, CsA was replaced by MMF and everolimus. VOD was diagnosed on d + 2 and graded moderate with weight gain (10%) and refractory thrombocytopenia with a high need for substitution. Progredient hepatomegaly was observed via ultrasound. Defibrotide was given from d + 2 for 24 days. The patient’s weight normalized with diuretic treatment within 1 week, while daily platelet transfusions were necessary for 3 weeks and hepatomegaly did not progress after 2 weeks.

VOD patient 5, a boy of nine years, was transplanted with allogeneic stem cells due to β-thalassemia major. He received busulfan, fludarabine, thiotepa, and Thymoglobulin^®^ as a conditioning regimen. He developed late-onset VOD on d + 51. At this time, the patient had already been discharged to the oncological day clinic. The symptoms of VOD were abdominal pain, ascites, newly emerged thrombocytopenia, and hyperbilirubinemia (total bilirubin: max. 1.9 mg/dl). Ultrasound detected hepatomegaly, reduced portal venous flow velocity (min. 10 cm/s), and early-stage cholecystitis. The patient received defibrotide for 28 days. The VOD was graded moderate due to intermittent renal insufficiency and oxygen requirement. After the initiation of defibrotide treatment, a platelet transfusion was necessary once. Portal venous flow and bilirubin levels normalized after 3 days of treatment, ascites diminished within 10 days, and hepatomegaly was regredient within 3 weeks.

**Fig. 1:**
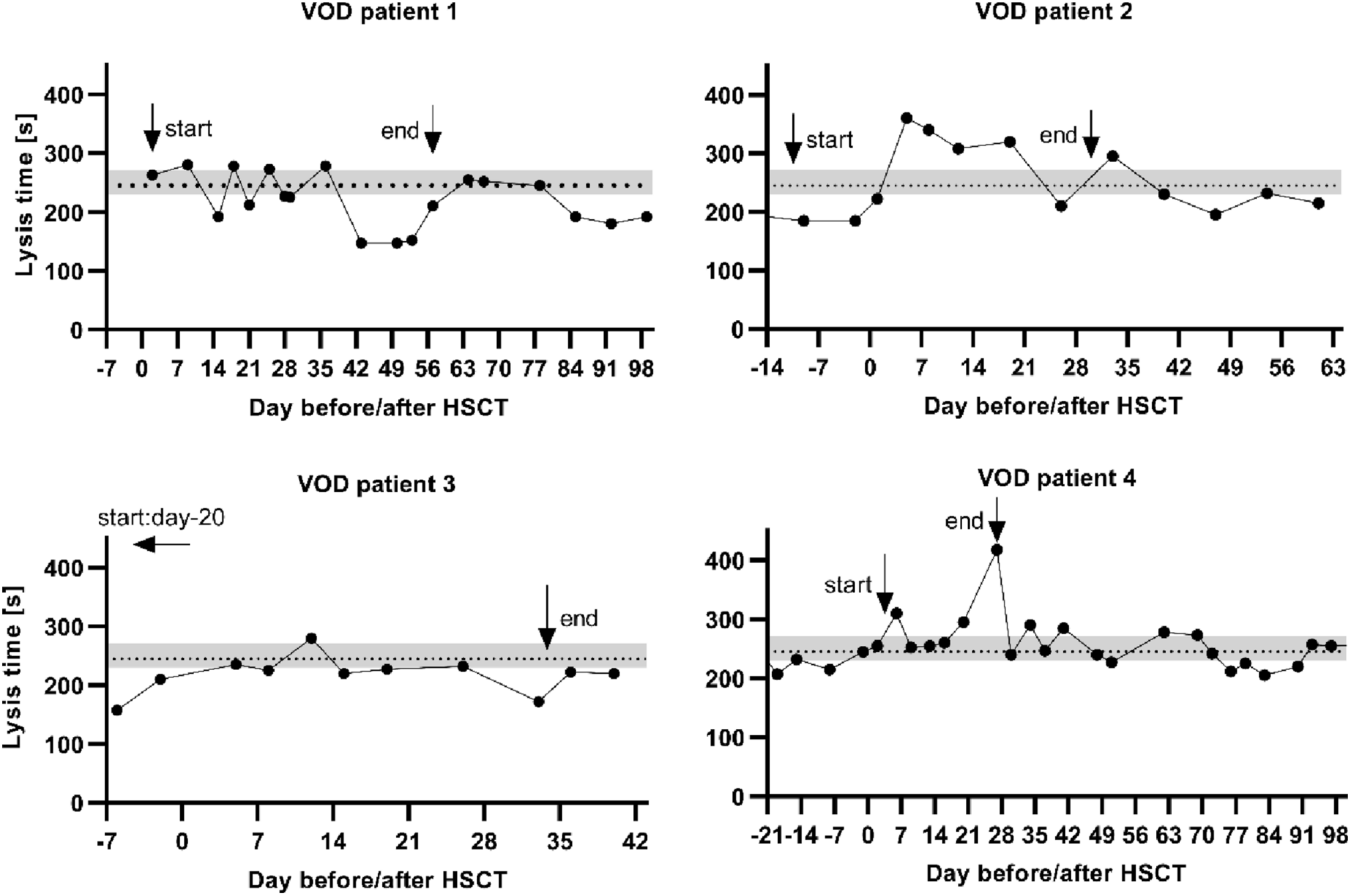
Lysis time of individual VOD patients. This graph shows the lysis time in TPA tests in seconds over time. Time is marked on the x-axis in days before (−) and days after ( +) HSCT (hematopoietic stem cell transplantation). The dotted line indicates the mean value and the grey area the 95% confidence interval for the lysis time in HSCT patients without VOD (veno-occlusive disease). The arrows show the beginning and the end of defibrotide treatment

Figure 1 shows the lysis time in TPA test over time (day before/after HSCT) for the VOD patients 1–4.

### Thrombelastography

For this analysis, the results of thrombelastography of the HSCT, VOD and control groups were compared.

Patients in the HSCT group exhibited a prolonged CT and reduced A05 and MCF compared to the control group (Table 2). No difference was observed between the VOD and HSCT groups.

**Table 2:** Thrombelastography measurements

Parameter	Control (*n* = 55)	d-7 to d-1					d 0 to d + 6				
		HSCT (*n* = *37*)	VOD (*n* = 4)	*p*: Ctrl vs. HSCT	*p*: Ctrl vs. VOD	*p*: HSCT vs. VOD	HSCT (*n* = 44)	VOD (*n* = 5)	*p*: Ctrl vs. HSCT	*p*: Ctrl vs. VOD	*p*: HSCT vs. VOD
EX test											
CT, s	45 ± 6.7	47 ± 8.1	53 ± 4.3	> 0.99	0.21	0.14	53 ± 7.2	58 ± 7.4	< 0.0001	0.0033	0.17
CFT, s	58 ± 27	80 ± 31	124 ± 110	0.0011	0.36	0.66	85 ± 65	67 ± 25	0.10	> 0.99	0.99
A05, mm	45 ± 5.6	37 ± 6.7	32 ± 9.3	< 0.0001	0.0129	0.30	36 ± 6.8	34 ± 2.7	< 0.0001	0.0017	0.32
MCF, mm	57 ± 4.5	50 ± 6.0	44 ± 10	< 0.0001	0.0072	0.22	49 ± 6.1	46 ± 3.6	< 0.0001	0.0006	0.13
ML, %	8.3 ± 3.2	6.3 ± 3.6	5.8 ± 3.1	0.06	0.98	0.87	4.9 ± 3.3	5.3 ± 2.6	< 0.0001	0.60	0.55
TPA test											
ML, %	95 ± 2.2	94 ± 1.6	93 ± 1.9	0.0353	0.05	0.15	92 ± 13	94 ± 0.89	0.0064	0.0408	0.17
LT, s	234 ± 50	300 ± 348	228 ± 30	> 0.99	> 0.99	0.84	251 ± 67	304 ± 57	> 0.99	0.13	0.06

Parameter	Control (*n* = 55)	d + 7 to d + 13					d + 14 to d + 21				
		HSCT (*n* = 45)	VOD (*n* = 5)	*p*: Ctrl vs. HSCT	*p*: Ctrl vs. VOD	*p*: HSCT vs. VOD	HSCT (*n* = 43)	VOD (*n* = 4)	*p*: Ctrl vs. HSCT	*p*: Ctrl vs. VOD	*p*: HSCT vs. VOD
EX test											
CT, s	45 ± 6.7	53 ± 6.7	65 ± 13	< 0.0001	0.0006	0.05	53 ± 7.2	60 ± 20	< 0.0001	0.08	0.28
CFT, s	58 ± 27	75 ± 52	77 ± 30	0.67	0.77	0.42	66 ± 36	79 ± 45	> 0.99	> 0.99	0.35
A05, mm	45 ± 5.6	36 ± 6.4	36 ± 8.5	< 0.0001	0.0331	0.68	38 ± 8.4	38 ± 8.2	< 0.0001	0.48	0.87
MCF, mm	57 ± 4.5	49 ± 6.1	48 ± 7.2	< 0.0001	0.0229	0.61	51 ± 7.1	50 ± 8.0	< 0.0001	0.41	0.86
ML, %	8.3 ± 3.2	3.9 ± 2.1	4.0 ± 2.6	< 0.0001	0.0382	0.99	4.1 ± 2.3	4.3 ± 2.6	< 0.0001	0.11	0.99
TPA test											
ML, %	95 ± 2.2	94 ± 1.6	93 ± 2.2	0.10	0.12	0.15	94 ± 1.6	95 ± 1.5	0.25	> 0.99	0.28
LT, s	234 ± 50	255 ± 70	278 ± 34	> 0.99	0.25	0.12	246 ± 53	330 ± 67	> 0.99	0.0299	0.0106

Thrombelastography parameters: mean value ± standard deviation

*A05* amplitude after 5 min, *CFT* clot formation time, *CT* coagulation time, *Ctrl* control, *HSCT *patients with hematopoietic stem cell transplant without VOD, *LT* lysis time, MCF – maximum clot firmness, *ML* maximum lysis, *mm* millimeter, *n* sample size, *p* probability value, *s* second, *VOD* patients diagnosed with veno-occlusive disease, *vs.* versus

**Fig. 2:**
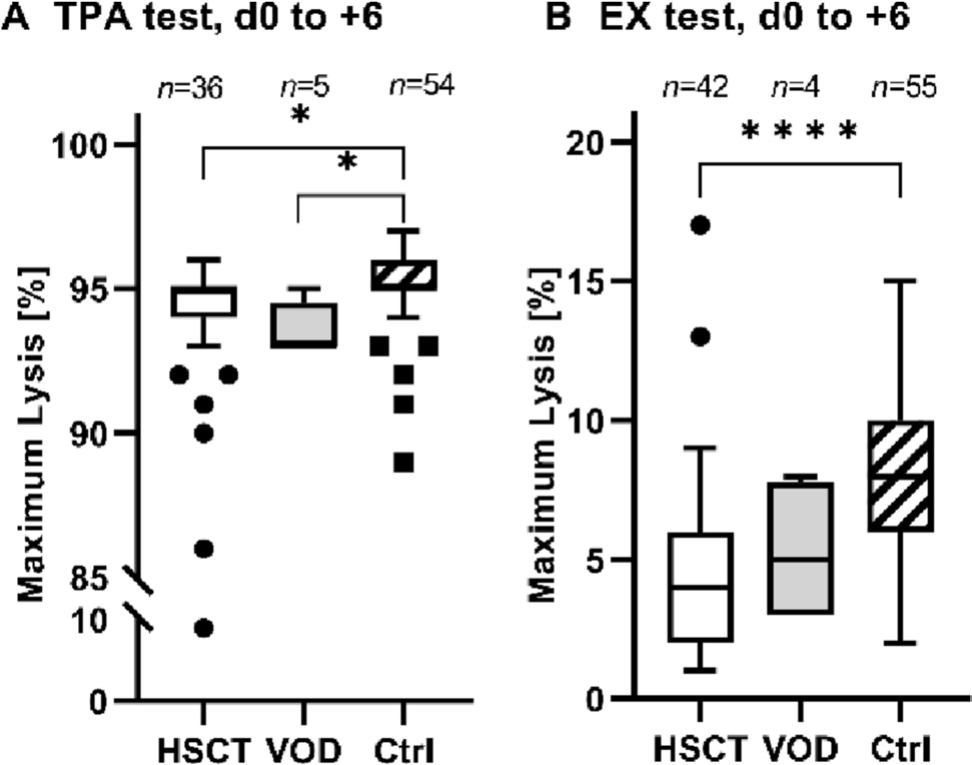
Maximum lysis of the HSCT, VOD and control groups. The graphs show the maximum lysis (in %) determined by the TPA test (**A**) and EX test (**B**) in the period of 0 to 6 days after HSCT (hematopoietic stem cell transplantation) in the HSCT, VOD (veno-occlusive disease), and control groups. Symbols indicate *p < 0.05, ****p < 0.0001, *n* indicates number of samples

The maximum lysis in the EX and TPA tests were lower in the HSCT group compared to the controls, although there was no difference between the VOD and HSCT groups (EX test: control: 8.3 ± 3.2%; HSCT: d0 to + 6: 5.3 ± 2.6%, *p* < 0.0001; d + 7 to + 13: 3.9 ± 2.1%, *p* < 0.0001; d + 14 to d + 21: 4.1 ± 2.3%, *p* < 0.0001; TPA test: control: 95 ± 2.2%; HSCT: d −7 to  − 1: 94 ± 1.6%, *p* = 0.0353; d0 to + 6: 92 ± 13%, *p* = 0.0064; Fig. 2).

**Fig. 3:**
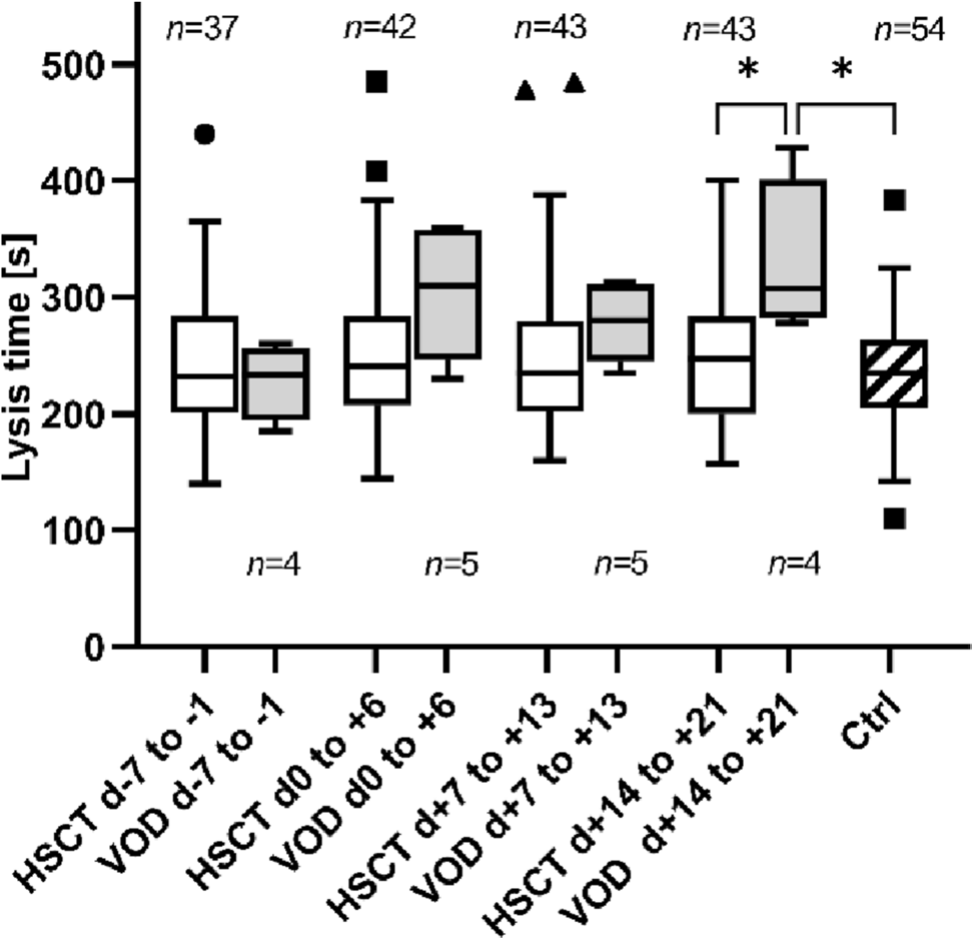
Lysis time of the HSCT, VOD and control groups. This graph shows the lysis time (in seconds) determined by the TPA test in the periods of − 7 to − 1 days before and 0 to + 6, + 7 to + 13 and + 14 to + 21 days after HSCT (hematopoietic stem cell transplantation) in the HSCT, VOD (veno-occlusive disease), and control groups. Symbol indicates *p < 0.05, *n* indicates number of samples

A significant difference was observed when comparing the VOD to the HSCT group. The lysis time after activation of fibrinolysis in the TPA test was prolonged in the VOD group at the time point of 3 weeks after HSCT d + 14 to + 21: VOD: 330 ± 67 s, HSCT: 246 ± 53 s, *p* = 0.0106; Fig. 3). Additionally, a significant difference between the VOD and control groups was observed in days + 14 to + 21 (control: LT 234 ± 50 s, *p* = 0.0299). For the time points of day 0 to + 6 and day + 7 and + 13 after HSCT lysis time was also prolonged in VOD patients, but without statistical significance (LT: d0 to + 6: VOD: 304 ± 57 s; HSCT: 251 ± 67 s, *p* = 0.06; d + 7 to + 13: VOD: 278 ± 34 s; HSCT: 255 ± 70 s, *p* = 0.12). Prolonged lysis time was not demonstrated at any of the measured time points for the stem cell transplanted patients without VOD.

### Correlation analyses

The results of thrombelastography were correlated with routine laboratory parameters, clinical symptoms, and risk factors of VOD. In all the following correlation analyses, HSCT and VOD group are analyzed together in one single group.

Maximum serum D-dimers correlated positively with LT in the TPA test (d0 to + 6: r = 0.32, *p* = 0.0348; d + 7 to + 13: r = 0.33, *p* = 0.0270) and negatively with ML in EX test (day + 14 to + 21: r = − 0.40, *p* = 0.0093). Elevated direct bilirubin inversely correlated with ML in the TPA test (d-7 to 0: r = − 0.3772, *p* = 0.0160), as did total serum bilirubin (TSB) (d0 to + 6: r = -0.58,* p* =  < 0.0001). Both parameters also correlated with LT (d0 to d + 6: direct bilirubin: r = 0.34, *p* = 0.0186, TSB: r = 0.31, *p* = 0.0331; d + 7 to + 13: direct bilirubin: r = 0.41, *p* = 0.0037, TSB: r = 0.30, *p* = 0.0372; d + 14 to d + 21: direct bilirubin: r = 0.44, *p* = 0.0180, TSB: r = 0.37, *p* = 0.0100). It was observed that LT correlated positively with weight gain (> 5%), progredient hepatomegaly, right upper quadrant pain and reduced portal venous flow velocity (d0 to d + 6: weight gain: r = 0.31, *p* = 0.0351; pain: r = 0.44, *p* = 0.0018; hepatomegaly: r = 0.37, *p* = 0.0108; d + 7 to + 13: pain: r = 0.35, *p* = 0.0143; hepatomegaly: r = 0.39, *p* = 0.0058; d + 14 to d + 21: weight gain: r = 0.42, *p* = 0.0035; pain: r = 0.37, *p* = 0.0103; hepatomegaly: r = 0.44, *p* = 0.0020; reduced portal venous flow velocity: r = 0.35, *p* = 0.0167). Additionally, there was a negative correlation of ML in TPA test with development of ascites and weight gain in day + 7 to + 13 (ML: ascites: r = -0.30; *p* = 0.0405; weight gain: r = − 0.31; *p* = 0.0333).

For the time of day 0 to + 6, negative correlations of busulfan-based conditioning for allogeneic grafts and of GvHD-prophylaxis with CsA/MTX with ML in the EX test were observed (busulfan-based conditioning: r = − 0.37, *p* = 0.0180; CsA/MTX: r = − 0.32,* p* = 0.0456).

A correlation between prolonged LT and allogeneic HSCT and leukemia also appeared (LT vs. allogeneic Tx: d0 to + 6: r = 0.31, *p* = 0.0344; d + 7 to + 13: r = 0.43,* p* = 0.0026; d + 14 to + 21: r = 0.39, *p* = 0.0065; LT vs. leukemia: d0 to + 6: r = 0.33, *p* = 0.0224; d + 7 to + 13: r = 0.29, *p* = 0.0422; d + 14 to + 21: r = 0.35, *p* = 0.0156).

## Discussion

While it has been shown that early diagnosis and initiation of treatment for VOD improves therapy response and survival, VOD is currently diagnosed by clinical symptoms mainly resulting from portal hypertension (Corbacioglu et al. 2018, 2004; Pescador et al. 2013). These symptoms appear when the disease is already established and early signs of developing VOD are not clinically apparent. The diagnosis is challenging as patients undergoing HSCT are at risk of developing other complications with overlapping symptoms. In VOD pathogenesis, the obstruction of hepatic sinusoids is accompanied by several other processes. Endothelial dysfunction, local inflammation with coagulation activation, and hypofibrinolysis play a role in the early development of VOD, as well as fibrosis of sinusoids resulting in sinusoidal obstruction (Cairo et al. 2020; Coppell et al. 2003; DeLeve et al. 1999). The aim of this study was to evaluate whether thrombelastography is a suitable tool to detect hypofibrinolysis in patients with VOD and whether it could complement established diagnostic criteria.

Endothelial dysfunction and local inflammation lead to activation of the endothelium via cytokines such as tumor necrosis factor or interleukin 1 beta. Endothelial activation results in an increase of plasminogen activator inhibitor-1 (PAI-1) (Milone et al. 2022). PAI-1 inhibits tissue plasminogen activator (tPA) and thus prevents activation of plasminogen and consequent fibrinolysis. Studies have shown that PAI-1 is increased in VOD patients while plasminogen levels are decreased (Lee et al. 2002; Park et al. 1997; Pihusch et al. 2005; Salat et al. 1999; Sartori et al. 2012). The highest PAI-1 levels were observed one to two weeks after HSCT (Pihusch et al. 2005). So far, this parameter is not part of routine testing in VOD diagnosis. High PAI-1 levels were shown to correspond with a prolonged lysis time in viscoelastic tests (Hammer et al. 2021). In this study, when activating lysis by recombinant tPA, patients suffering from VOD had a longer LT compared to HSCT patients.

Although there was no difference in baseline values before HSCT, LT at day + 14 to + 21 was significantly prolonged at 330 ± 67 s in the presence of VOD compared to the HSCT patients without VOD (*p* = 0.0106) and compared to the control group (*p* = 0.0299) (Fig. 3). In comparison, however, the ML of the VOD patients in the TPA test was not significantly reduced compared to the control group and the HSCT patients without VOD. While for the time periods between day 0 to + 6 and day + 7 to + 13 a tendency towards longer lysis time was also observed in the VOD group compared to the HSCT and control group, although not statistically significant, this could not be demonstrated for the stem cell transplanted patients without VOD at all time points, which allows a good differentiation from the VOD patients.

The prolonged LT is a sign of hypofibrinolysis in VOD patients. Lysis time in the TPA test could be a rapidly and easily measured marker of hypofibrinolysis in patients with suspected VOD, since the results are available within minutes. High-risk patients could thus be screened several times a week.

Additionally, a positive correlation of maximum serum D-dimers with LT was observed. Although D-dimers are known for their high negative predictive value in ruling out thrombosis, they lack sensitivity for the diagnosis of thrombosis. Several studies have detected elevated D-dimers in VOD patients (Jevtic et al. 2011; Park et al. 1997; Sartori et al. 2012). Although elevated D-dimer levels are not specific enough for a diagnosis of VOD, pooling PAI-1-, D-dimer-, and bilirubin levels predicts VOD (Sartori et al. 2012).

Spontaneous lysis was lower in the HSCT group compared to the control group. ML was significantly reduced in the EX test in the HSCT (d 0 to + 6, d + 7 to + 13, d + 14 to + 21) and VOD group (d + 7 to + 13) compared to the control group, while differences between HSCT or VOD and control group were not so consistent regarding ML in TPA test. Bachler et al. explained discrepancies between ML results in EX and TPA test by a decreased response to recombinant tPA which could be due to the presence of PAI-1 (Bachler et al. 2021). Even though there was no significant difference between the VOD and HSCT groups, this can be a sign of reduced fibrinolytic capacity in patients undergoing HSCT, which puts them at risk of developing VOD. ML correlated inversely with bilirubin levels, thereby suggesting a connection between reduced fibrinolytic capacity and hyperbilirubinemia. Another study did not find significant differences in maximum lysis when comparing HSCT patients with and without VOD (Rupa-Matysek et al. 2017). Other data comparing fibrinolytic capacities in HSCT patients to healthy controls are scarce.

Defibrotide treatment has been proven to be an efficient therapy for VOD, but the exact mechanisms of function for this drug are not completely understood (Mohty et al. 2021; Richardson et al. 2016). Defibrotide acts as an anti-inflammatory agent, reduces endothelial adhesion, has antithrombotic and thrombolytic properties and reduces vascular tone (Pescador et al. 2013). Although the number of cases developing VOD in this cohort is small (*n* = 5), some observations can be deducted from the thrombelastography measurements obtained. LT did not immediately normalize after initiation of treatment in VOD patients but was still significantly prolonged 3–4 weeks after HSCT. It approximated the mean value of LT in other HSCT patients around the time that clinical symptoms resolved, and treatment was subsequently discontinued. This corresponds to previously published data that show decreasing, but still elevated PAI-1 levels up to 3 weeks after diagnosis in patients responding to defibrotide treatment (Kaleelrahman et al. 2003; Sartori et al. 2012). Looking at several examples of VOD patients in this study (Fig. 1), a prolonged lysis time especially in the first week after HSCT can be observed, although in two patients (VOD patients 2 and 3), defibrotide treatment had been initiated several days earlier. This could represent a “peak” hypofibrinolysis in the first week after HSCT. These observations should be further investigated in a larger set-up.

Correlating symptoms of VOD with thrombelastography measurements, the analyses show an association of prolonged LT with the occurrence of hepatomegaly, right upper quadrant pain, weight gain, and a reduced portal venous flow velocity in ultrasound examinations. This underlines that a correlation between established diagnostic criteria and symptoms of VOD exists for LT in the TPA test and that this could be an additional diagnostic tool.

Correlations of impaired lysis with known risk factors for the development of VOD were analyzed. Among many other characteristics, young age, leukemia as an underlying disease, allogeneic graft, type of conditioning regimen, GVHD prophylaxis, and previous treatment have been identified as risk factors so far (Barker et al. 2003; Corbacioglu et al. 2019; Dalle and Giralt 2016). The odds ratio for developing VOD under busulfan-based versus other conditioning regimens is 2.6–4.5 (Dalle and Giralt 2016). Hepatotoxicity is attributed to busulfan because it depletes glutathione in hepatocytes, thereby increasing oxidative stress (DeLeve et al. 2000). The standard immunosuppression for GvHD prophylaxis in allogeneic HSCT is CsA and MTX and in the studied cohort, 45% of patients received this combination. An odds ratio of 3.3 for the development of VOD in pediatric patients when using CsA and MTX compared to other immunosuppression was described (Barker et al. 2003). CsA leads to endothelial dysfunction and is also a risk factor for other endothelium-mediated transplant-related complications such as thrombotic microangiopathy or posterior reversible encephalopathy syndrome (Milone et al. 2022). CsA affects fibrinolysis by increasing PAI-1 and decreasing tPA (Malyszko et al. 1996).

This study observed a correlation of longer lysis time with patients receiving allogeneic grafts and with patients diagnosed with leukemia. A lower maximum lysis correlated with the use of busulfan-based conditioning regimens for allogeneic grafts and of CsA and MTX as GvHD prophylaxis. Thus, patients undergoing allogeneic HSCT for leukemia with busulfan-based conditioning and CsA as GvHD prophylaxis can be identified as patients with impaired lysis. Patients accumulating these risk factors might profit from close monitoring via thrombelastography and even defibrotide prophylaxis.

## Conclusion

In summary, thrombelastography can be used as a supportive diagnostic tool in the course of VOD due to the occurrence of hypofibrinolysis in all patients with VOD after HSCT.

Larger studies are needed to evaluate the exact onset of hypofibrinolysis and determine specific cut-off-values. Thrombelastography might be a useful tool to identify and monitor high-risk patients or support diagnosis of VOD.

## Data Availability

The data that support the findings of this study are available from the corresponding author upon reasonable request.
